# Mortality in sepsis among patients with gastrointestinal malignancies: a nationwide analysis 2017–2021

**DOI:** 10.62838/jccm-2026-0056

**Published:** 2026-10-30

**Authors:** Barath Prashanth Sivasubramanian, Diviya Bharathi Ravikumar, Husna Qadeer, Abul Hasan Shadali Abdul Khader, Rhea Prasad, Sindhu Chandra Pokhriyal, Tenzin Tamdin, Deepak Venkataraman, Viraj Panchal, Vikramaditya Samala Venkata, Raghavendra Tirupathi, Rutul Dalal

**Affiliations:** Department of Internal Medicine, Northeast Georgia Medical Center, Gainesville, Georgia, USA; Department of Internal Medicine, ESIC MC and PGIMSR, Chennai, Tamil Nadu, India.; Department of Internal Medicine, Government Kilpauk Medical College, Chennai, Tamil Nadu, India; Interfaith Medical Center - One Brooklyn Health, Brooklyn, New York, USA; Department of Internal Medicine, Danbury Hospital, Affiliate Yale School of Medicine, Danbury, Connecticut, USA; Davao Medical School Foundation Inc., Philippines.; Department of Medicine Smt. N.H.L. Municipal Medical College and SVPISMR, Ahmedabad, Gujarat, India.; Physician at Cheshire Medical Center/Dartmouth Health Keene, NH, USA; Keystone health, Chambersburg, Pennsylvania, USA; MD, FACP, FRCP, FIDSA, Infectious Diseases, Penn State of Health, St Joseph Medical Center, Pennsylvania, USA

**Keywords:** gastrointestinal, gastric, cancer, malignancies, sepsis, hepatocellular.

## Abstract

**Background:**

Sepsis is a leading cause of morbidity and mortality in patients with gastrointestinal (GI) malignancies, with in-hospital mortality rates of 19.8% to 28%, significantly higher than in non-cancer patients. While sepsis outcomes have been extensively studied in the general population, large-scale population-level analyses specific to GI cancers remain limited. This study aimed to identify the outcomes of sepsis in gastric, colon, hepatic, and pancreatic cancers in the United States.

**Methods:**

We conducted a retrospective analysis of the National Inpatient Sample (2017–2021) using ICD-10 codes, including adults ≥18 years with gastric (n=205,599), colon (n=851,120), hepatocellular (n=433,145), or pancreatic (n=557,204) cancer. Propensity matching and multivariate regression were used to adjust for confounders. A p-value of ≤0.05 was considered significant.

**Results:**

Sepsis prevalence was 13.79% in gastric, 12.6% in colon, 18.76% in hepatocellular, and 19.22% in pancreatic cancer hospitalizations. In-hospital mortality was significantly higher with sepsis across all four malignancies (gastric 23.57% vs. 5.09%; colon 17.72% vs. 2.74%; hepatocellular 22.34% vs. 6.22%; pancreatic 17.26% vs. 5.11%). After propensity matching and adjustment, sepsis was independently associated with increased mortality in gastric (aOR 1.87), colon (aOR 2.39), hepatocellular (aOR 1.60), and pancreatic cancer (aOR 1.43) (all p<0.001). Mortality among patients requiring ICU-level care was 70.28%, 70.88%, 72.97%, and 67%, respectively. Acute kidney injury, acute respiratory failure, invasive ventilation, disseminated intravascular coagulation, and shock were consistent mortality predictors across all malignancies (p<0.05). Sepsis was also associated with significantly higher hospitalization charges and longer length of stay (p<0.001).

**Conclusion:**

Our study revealed that patients with gastrointestinal malignancies experience high mortality rates when affected by sepsis, with acute organ dysfunction and ICU-level care as key predictors of poor outcomes. These findings highlight GI cancer as a high-risk population for adverse sepsis outcomes and support the need for prospective studies evaluating early recognition and guideline-directed therapies.

## Introduction

GLOBOCAN data indicate that gastrointestinal cancers contribute substantially to the global cancer burden, with gastric cancer accounting for 5.7% of new cases every year and 8.2% of cancer-related deaths, pancreatic 2.5% and 4.5%, hepatic 4.7% and 8.2%, and colon cancer 6.1% and 5.8%, posing a significant public health challenge worldwide [1]. The common complications encountered in patients with GI malignancies include acute respiratory failure, acute kidney injury, myocardial infarction, and acute heart failure. These complications are further exacerbated by age, gender, and underlying comorbidities, contributing to in-hospital mortality [2].

Oncological patients account for up to 20% of ICU admissions, with sepsis as the leading cause of admission [3]. Sepsis is one of the leading causes of morbidity and mortality in patients with GI malignancies, with in-hospital mortality rates reaching approximately 19.8% to 28%, significantly higher than in non-cancer patients with sepsis and up to 40% for septic shock, significantly higher than in non-cancer patients [4,5]. According to previous studies, individuals with cancer are ten times more likely to develop sepsis compared to those without cancer [6], primarily because of factors such as immunosuppression from the underlying disease or certain anti-cancer treatments, as well as invasive procedures like tunneled catheters, urinary catheters, ports, and drainages [7].

While sepsis outcomes have been extensively studied in the general population, large-scale population-level analyses of sepsis specifically in patients with GI malignancies remain limited [8,9,10,11]. Most existing studies examining mortality, hospital costs, and healthcare utilization in this subpopulation are single-center analyses with small sample sizes, limiting generalizability [4,12]. Prior population-based studies have relied on administrative data but lacked information on end-of-life care and healthcare resource utilization in this group [13].

We conducted a nationwide retrospective analysis comparing in-hospital outcomes with GI malignancies with sepsis versus those without sepsis. Our primary objective was to compare in-hospital mortality between these two groups and identify factors independently associated with mortality among patients with GI malignancies and sepsis. Additionally, we aimed to identify the length of stay and total hospital charges in these patients.

## Materials and Methods

### Design and Data Source

The 2017–2021 National Inpatient Sample (NIS) database was used for this cross-sectional study. The Healthcare Cost and Utilization Project (HCUP) is sponsored by the Agency for Healthcare Research and Quality (AHRQ) and is responsible for NIS [14,15]. Its objective was to produce estimates of inpatient utilization, expenditures, and outcomes at the regional and national levels for the United States. Variables provided by NIS include patient demographics (including sex and age), diagnosis and procedure codes obtained from the Clinical Modification/Procedure Coding System (ICD-10-CM/PCS), severity and comorbidity assessments, hospital attributes, discharge status, and length of hospitalization.

### Ethical Consideration, Sample Size, and Study Population

The NIS is a publicly accessible database that has been de-identified. An institutional review board was not required for approval. In this investigation, no predetermined sample size was calculated. Adults aged 18 and above admitted with an ICD-10-CM code of gastrointestinal malignancies (gastric, pancreatic, hepatic, and colonic malignancies) were included. ICD-10-CM/PCS codes were used to identify patients and are provided in the supplementary file Table 1. The ICD-10 codes of sepsis have a specificity of 98% (95% CI 98–99%) and a median positive predictive value of 72% based on a recent meta-analysis (Liu et al. 2022). However, the study also reported a sensitivity of 35% reflects undercoding of sepsis (Liu et al. 2022). We ensured adherence to the data user agreement of the AHRQ before accessing the NIS databases. The databases employed comply with the Privacy Rule of the Health Insurance Portability and Accountability Act (HIPAA) and contain no explicit patient identifiers, as defined by HIPAA. All non-malignant patients and pregnant individuals were excluded from the study. Figure 1 describes the population of the study.

**Figure 1.**
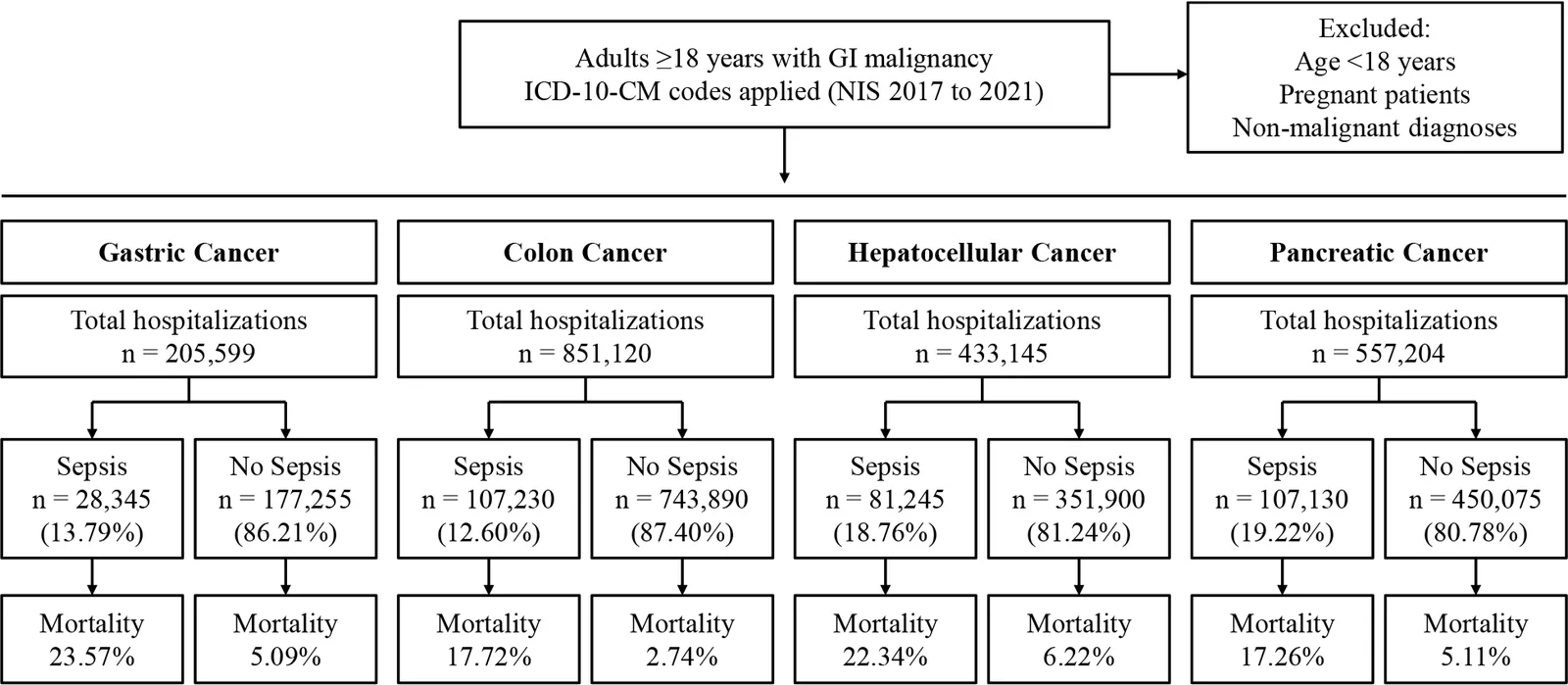
Study Population: NIS 2017 to 2021, Gastrointestinal Malignancies

### Variables Assessed

The categories of gender were male and female. The patient’s racial categories included White (referential), African American, Hispanic, Asian, Pacific Islander, and Native American. Medicare, Medicaid, private insurance, and other forms of payment (including self-pay/no charge) were used to determine insurance status. Comorbidities and complications such as diabetes mellitus, hypertension, acute respiratory distress syndrome (ARDS), acute kidney injury, and vasopressor usage are all included in the codes. The codes are detailed in Table 1 of the supplementary file. The in-patient mortality, total hospital charges, and length of stay were obtained from the NIS database using the codes for ‘DIED’, ‘TOTCHG’, and ‘LOS’, respectively. ICU level of care was determined by the presence of acute kidney injury requiring hemodialysis, vasopressor support, invasive ventilation, and shock.

**Table 1:** Mortality, palliative care, and hospitalization resources of patients with gastrointestinal malignancies

**Cancer Type**	**Palliative Care Utilization (Sepsis% vs. Non-Sepsis%)**	**Mortality Rate (Sepsis% vs. Non-Sepsis%)**	**Adjusted Odds Ratio for Mortality (95% CI)**	**Hospital Charges, Coefficient (95% CI)**	**Length of Stay, Coefficient (95% CI)**
Stomach Cancer	29.85% vs. 15.91%	23.57% vs. 5.09%	1.87 (1.66–2.10)	$22,469.32 ($17,077.59–$27,861.06)*	2.35 (1.98–2.71) days*
Colon Cancer	22.55% vs. 8.41%	17.72% vs. 2.74%	2.39 (2.22–2.59)	$18,017.33 ($15,608.34–$20,426.32)*	2.37 (2.22–2.52) days*
Hepatocellular Cancer	28.12% vs. 17.04%	22.34% vs. 6.22%	1.60 (1.50–1.73)	$15,725.13 ($12,379.23–$19,071.03)*	1.93 (1.76–2.09) days*
Pancreas Cancer	26.96% vs. 17.93%	17.26% vs. 5.11%	1.43 (1.34–1.54)	$9,351.10 ($7,410.74–$11,291.46)*	1.23 (1.11–1.36) days*

Legend: Mortality risk model was run by performing a propensity matched analysis with age over 60 years and gender and adjusted with age over 60 years, gender, race, size of the hospital, acute organ dysfunction (acute respiratory failure, acute kidney injury, infective endocarditis, metabolic encephalopathy, hepatic dysfunction), comorbidities (diabetes, anemia), and complications (invasive ventilation, disseminated intravascular coagulation, shock, and ICU-level care).

### Outcomes

The primary objective of our study is to determine the factors associated with in-hospital mortality among adults with gastrointestinal malignancies (gastric, pancreatic, hepatic, and colonic malignancies) and a diagnosis of sepsis. The secondary objective included comparing the duration and the total costs associated with the hospital stay.

### Statistical Methods

We analyzed continuous variables with means and t-tests, and qualitative variables with the chi-square test. We set a significance level of p ≤ 0.05. Stata v18 was used to conduct the analysis. We used two different methods to adjust for confounders in our analysis: Propensity score matching and multivariate regression analysis. Propensity scores were used to match Gastrointestinal malignancies (gastric, pancreas, liver, and colon) with sepsis. A nonparsimonious multivariate logistic regression model was developed to estimate the propensity score for developing mortality using the following variables: age over 60 years and gender. The double robust method was then used to generate treatment weights and the inverse probability of treatment weighting was utilized to match cases with controls using generalized linear models [16]. The second analysis used multivariable regression models to adjust the results for potential confounders. Multivariable regression models were constructed using all confounders that were found to be significantly associated with the outcome in the univariate analysis, with a cutoff p-value of 0.1. Variables deemed important determinants of the outcomes based on the literature were forced into the models. Variables that could contribute to confounding were further analyzed using interaction analyses.

## Results

### Impact of Sepsis in Gastric Malignancy

Among 205,599 hospitalizations for gastric cancer, 13.79% were admitted with sepsis. In-hospital mortality was substantially higher among patients with sepsis compared to those without sepsis (23.57% vs. 5.09%, p≤0.001). After propensity score matching and multivariable adjustment, sepsis remained independently associated with mortality (aOR 1.87, p≤0.001), with incremental hospitalization charges of $22,469 (p≤0.001) and an additional length of stay of 2.35 days (p≤0.001).

Patients with sepsis were marginally older than those without sepsis (mean age 65.84 vs. 65.82 years). By race, White patients comprised a smaller proportion of the sepsis group than the non-sepsis group, while African American patients comprised a larger proportion.

Among patients with gastric cancer and sepsis, acute organ dysfunction and complications independently predicted mortality. Hepatic dysfunction conferred the highest organ-failure-related risk (OR 3.34), followed by acute kidney injury (OR 2.17) and metabolic encephalopathy (OR 1.56) (all p≤0.05). Among complications, shock (OR 6.86, p≤0.05), invasive ventilation (OR 4.91), acute respiratory failure (OR 3.14), and ICU-level care (OR 2.57) were significantly associated with mortality. Table 2 and supplementary table 2 detail mortality predictors in gastric malignancy.

Figures 2 and 3 display palliative care utilization and in-hospital mortality by cancer type. Figure 4 shows sepsis-associated mortality across GI cancers, and Figures 5A–5B show the incremental charges and length of stay associated with sepsis in gastric cancer.

**Table 2:** Factors Associated with mortality in gastric cancer

	**Sepsis % (n=28,345)**	**Non-Sepsis % (n=177,255)**	**Mortality % (n=6,680)**	**Adjusted OR (95% CI)**	**p-value**
Sociodemographics					
Age Over 60 years	69.69	68.35	72.46	1.05 (0.92–1.20)	0.48
Gender					
Male	64.7	63.04	65.34	Reference	Reference
Female	35.3	36.96	34.66	1.16 (1.03–1.31)	0.01
Race					
White	51.33	53.36	48.51	Reference	Reference
Black	17.48	16.41	18.16	1.08 (0.93–1.27)	0.30
Hispanic	18.6	17.66	19.46	1.28 (1.09–1.50)	0.002
Asian or Pacific Islander	7.79	7.81	8.05	1.31 (1.05–1.64)	0.02
Native American	0.7	0.72	0.31	0.75 (0.35–1.59)	0.45
Other	4.1	4.05	5.52	1.51 (1.12–2.03)	0.007
Hospital size according to the number of beds					
Small	16.9	16.04	16.47	1.19 (1.01–1.40)	0.04
Medium	25.54	25.77	27.25	1.14 (1.00–1.31)	0.05
Large	57.56	58.18	56.29	Reference	Reference
Comorbidites					
Diabetes	27.99	26.87	26.12	0.84 (0.74–0.96)	0.01
Anemia	51.53	43.25	43.49	0.79 (0.70–0.88)	<0.001
Acute Organ Dysfunction					
Acute Kidney Injury	42.65	16.52	65.72	2.17 (1.91–2.46)	<0.001
Infective Endocarditis	2.7	0.52	<5	0.45 (0.27–0.77)	0.004
Metabolic Encephalopathy	11.1	2.27	18.64	1.56 (1.27–1.92)	<0.001
Hepatic Dysfunction	5.8	1.36	14.52	3.34 (2.61–4.27)	<0.001
Myocardial Infarction	20.29	19.57	22.31	0.90 (0.77–1.04)	0.16
Complications					
Acute Respiratory Failure # Invasive Ventilation					
Acute Respiratory Failure	32.1	6.92	57.71	3.14 (2.71–3.65)	<0.001
Invasive Ventilation	14.92	1.88	38.25	4.91 (3.55–6.80)	<0.001
Acute Respiratory Failure and Invasive Ventilation	-	-	-	5.24 (4.18–6.57)	<0.001
Disseminated Intravascular Coagulation # Shock					
Disseminated Intravascular Coagulation	<5	<5	5.01	1.10 (0.86–1.41)	0.44
Shock	31.98	<5	61	6.86 (3.84–12.26)	<0.001
Disseminated Intravascular Coagulation and Shock	-	-	-	1.33 (0.71–2.51)	0.37
ICU-level care	36.69	<5	70.28	2.57 (1.96–3.36)	<0.001

Legend: A dash (–) in the frequency columns indicates that the variable was constructed as an interaction term used exclusively for confounder adjustment in the mortality model and does not have a discrete prevalence estimate applicable to the descriptive cohort.

**Supplementary Table 2:** Factors associated with mortality in gastric cancer

	**Sepsis % (n=28,345)**	**Non-Sepsis % (n=177,255)**	**Mortality % (n=6,680)**
Comorbidities			
Arrhythmia	10.92	7.89	13.4
Chronic Kidney Disease	11.43	8.74	13.32
Obesity	3.86	3.25	3.97
Palliative Care	29.85	15.91	54.34
Infections			
Gram-Negative Sepsis	5.87	-	6.44
E. coli	5.57	-	<5
Enterococcus	3.74	-	<5
Pseudomonas	<5	-	<5
S. aureus	4.92	-	<5

Legend: A dash (–) in the column indicates that the variable was not studied in the non-sepsis cohort.

**Figure 2.**
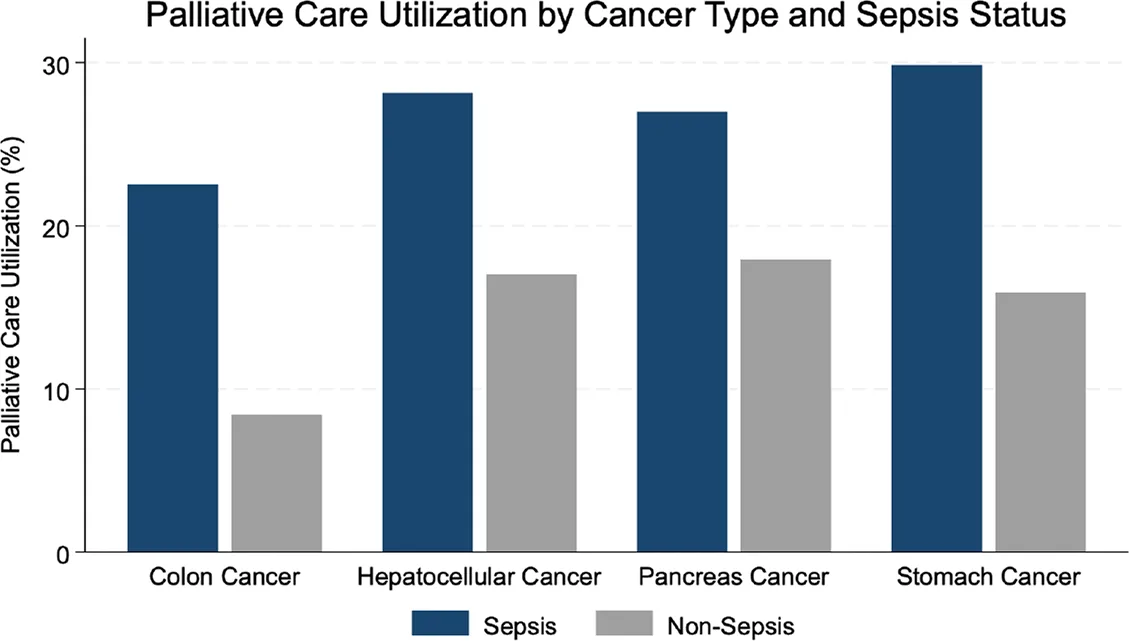
Palliative care utilization by cancer type and sepsis status Legend: Bar graph depicting unadjusted descriptive palliative care utilization rates by cancer type and sepsis status. Palliative care consultation was more frequently utilized among patients with sepsis compared with those without sepsis across all cancers, including stomach cancer (29.85% vs. 15.91%), colon cancer (22.55% vs. 8.41%), hepatocellular cancer (28.12% vs. 17.04%), and pancreatic cancer (26.96% vs. 17.93%).

**Figure 3.**
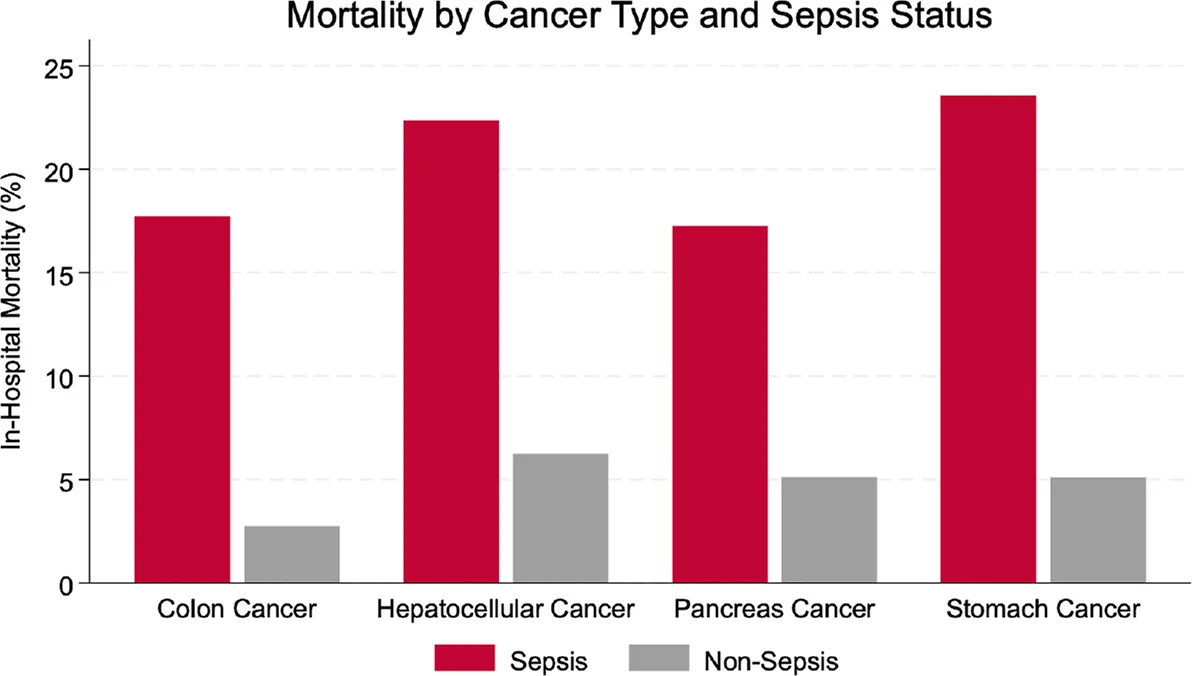
In-Hospital mortality rates by cancer type and sepsis status Legend: Bar graph showing unadjusted descriptive in-hospital mortality rates stratified by sepsis status across gastrointestinal cancer types. Mortality among patients with sepsis was higher than among those without sepsis for all cancers, including stomach cancer (23.57% vs. 5.09%), colon cancer (17.72% vs. 2.74%), hepatocellular cancer (22.34% vs. 6.22%), and pancreatic cancer (17.26% vs. 5.11%).

**Figure 4.**
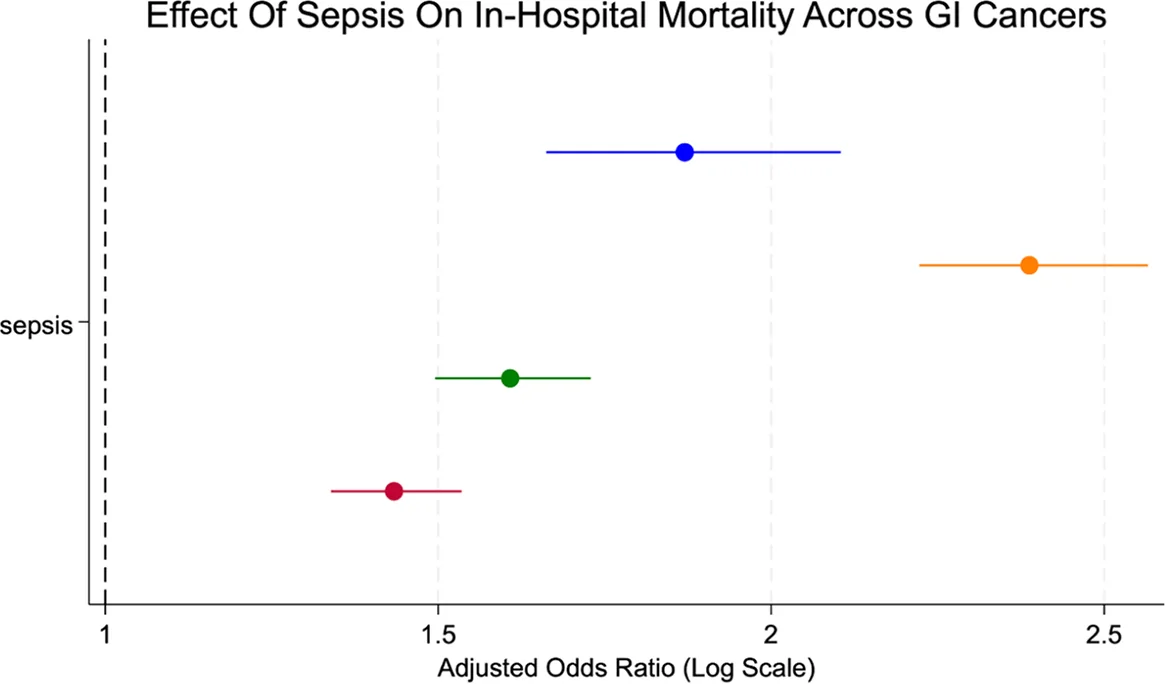
In-Hospital Mortality Associated With Sepsis Across Gastrointestinal Cancers Legend: Forest plot displaying adjusted odds ratios with 95% confidence intervals for in-hospital mortality comparing hospitalizations with sepsis versus without sepsis across gastrointestinal cancers. Colon cancer is shown in orange (adjusted OR 2.39, 95% CI 2.22–2.59), stomach cancer in blue (adjusted OR 1.87, 95% CI 1.66–2.10), hepatocellular cancer in green (adjusted OR 1.60, 95% CI 1.50–1.73), and pancreatic cancer in red (adjusted OR 1.43, 95% CI 1.34–1.54). Estimates were derived from propensity score–matched cohorts and multivariable logistic regression models adjusted for age >60 years, sex, race, hospital bed size, acute organ dysfunction (acute respiratory failure, acute kidney injury, infective endocarditis, metabolic encephalopathy, hepatic dysfunction), comorbidities (diabetes mellitus, anemia), and in-hospital complications (invasive mechanical ventilation, disseminated intravascular coagulation, shock, and ICU-level care). The vertical reference line indicates an adjusted odds ratio of 1.0.

**Figure 5A.**
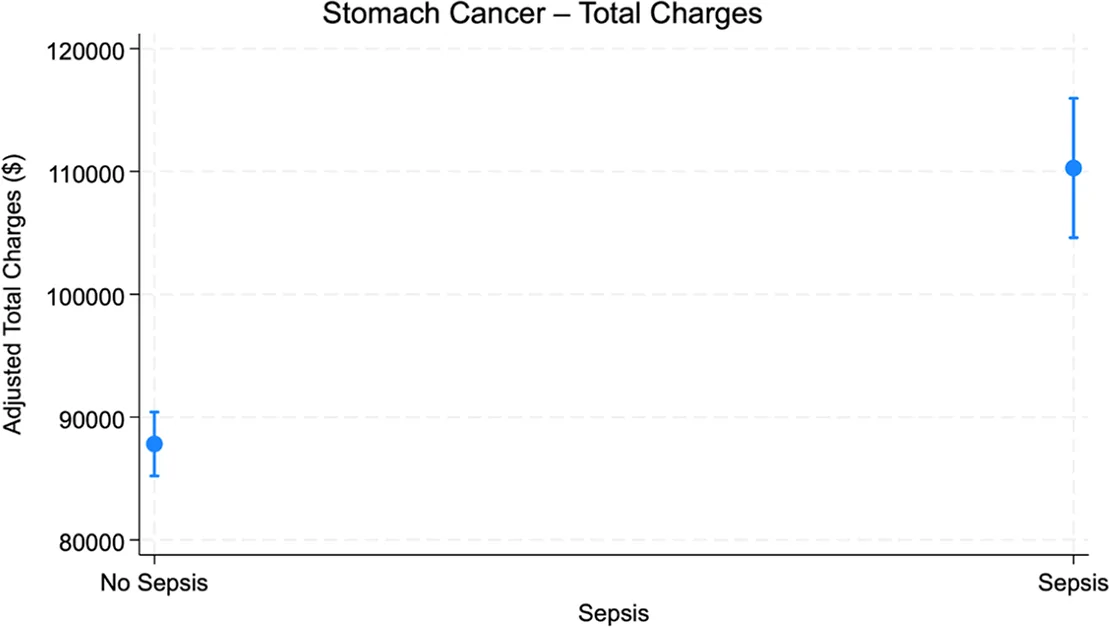
Total hospitalization charges associated with sepsis in stomach cancer Legend: Difference in total hospitalization charges comparing sepsis versus non-sepsis hospitalizations among patients with stomach cancer. Sepsis was associated with an increase in hospital charges of $22,469.32 (95% CI $17,077.59–$27,861.06), estimated using propensity score–matched cohorts and multivariable linear regression adjusted for demographic characteristics, hospital factors, acute organ dysfunction, comorbidities, and in-hospital complications.

**Figure 5B.**
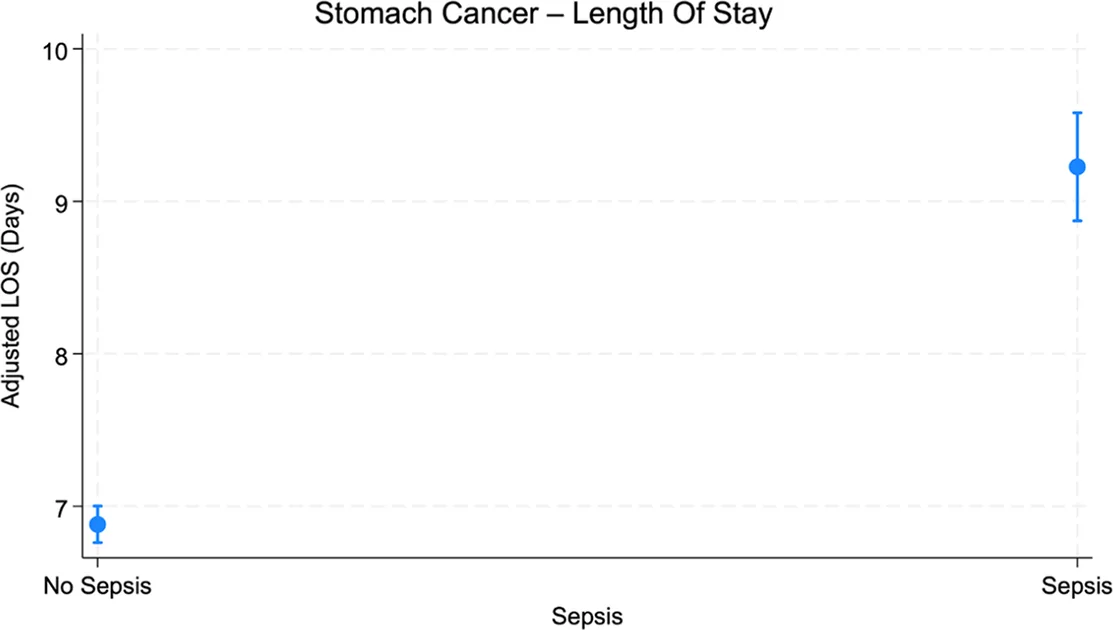
Length of stay associated with sepsis in stomach cancer Legend: Difference in hospital length of stay comparing sepsis versus non-sepsis hospitalizations among patients with stomach cancer. Sepsis was associated with an increased length of stay of 2.35 days (95% confidence interval 1.98–2.71), based on propensity score–matched cohorts with multivariable adjustment.

### Impact of Sepsis in Colonic Malignancy

Among 851,120 hospitalizations for colon cancer, 12.60% were admitted with sepsis. In-hospital mortality was markedly higher with sepsis than without sepsis (17.72% vs. 2.74%, p≤0.001). After propensity matching and adjustment, sepsis remained independently associated with mortality (aOR 2.39, p≤0.001), incremental charges of $18,017 (p≤0.001), and an additional length of stay of 2.37 days (p≤0.001).

Patients with sepsis were slightly older than those without sepsis (mean age 67.51 vs. 67.24 years). Females comprised a smaller proportion of the sepsis cohort than the non-sepsis cohort. By race, White patients comprised a smaller proportion of the sepsis group than the non-sepsis group, while African American patients comprised a larger proportion.

Among patients with colon cancer and sepsis, hepatic dysfunction conferred the highest organ-failure-related mortality risk (OR 3.57), followed by acute kidney injury (OR 1.68) and metabolic encephalopathy (OR 1.39). Among complications, invasive ventilation (OR 6.46), shock (OR 6.25), acute respiratory failure (OR 3.31), ICU-level care (OR 2.00), and disseminated intravascular coagulation (OR 1.50) (all p≤0.05) were each independently associated with mortality. Table 3 and supplementary table 3 detail mortality predictors in colon malignancy.

**Table 3:** Factors Associated with mortality in colon cancer

	**Sepsis % (n=107,230)**	**Non-Sepsis % (n=743,890)**	**Mortality % (n=18,990)**	**Adjusted OR (95% CI)**	**p-value**
Sociodemographics					
Age Over 60 years	72.65	70.43	79.81	1.39 (1.27–1.51)	<0.001
Gender					
Male	55.35	50.42	54.56	Reference	Reference
Female	44.65	49.58	45.44	1.08 (1.01–1.16)	0.024
Race					
White	66.55	70.64	65.87	Reference	Reference
Black	15.39	13.6	16.68	1.09 (0.99–1.21)	0.089
Hispanic	10.76	9.2	9.43	0.99 (0.87–1.12)	0.831
Asian or Pacific Islander	<5	<5	<5	1.14 (0.94–1.37)	0.178
Native American	<5	<5	<5	0.89 (0.51–1.55)	0.674
Other	<5	<5	<5	1.25 (1.00–1.55)	0.049
Hospital size according to the number of beds					
Small	20.58	20.26	19.88	1.02 (0.92–1.12)	0.744
Medium	29.6	28.36	29.78	1.01 (0.93–1.09)	0.888
Large	49.82	51.38	50.34	Reference	Reference
Comorbidites					
Diabetes	29.61	26.24	27.01	0.81 (0.75–0.87)	<0.001
Anemia	51.02	38.05	43.76	0.84 (0.78–0.90)	<0.001
Acute Organ Dysfunction					
Acute Kidney Injury	45.89	15.47	67.61	1.68 (1.55–1.81)	<0.001
Infective Endocarditis	<5	<5	<5	0.56 (0.43–0.75)	<0.001
Metabolic Encephalopathy	12.13	<5	20.14	1.39 (1.24–1.55)	<0.001
Hepatic Dysfunction	5.26	<5	14.93	3.57 (3.09–4.13)	<0.001
Myocardial Infarction	20.87	18.56	24.86	1.07 (0.98–1.16)	0.142
Complications					
Acute Respiratory Failure # Invasive Ventilation					
Acute Respiratory Failure	27.62	5.38	58.03	3.31 (3.02–3.63)	<0.001
Invasive Ventilation	12.1	<5	36.33	6.46 (5.29–7.88)	<0.001
Acute Respiratory Failure and Invasive Ventilation	-	-	-	5.33 (4.68–6.06)	<0.001
Disseminated Intravascular Coagulation # Shock					
Disseminated Intravascular Coagulation	<5	<5	<5	1.50 (1.29–1.75)	<0.001
Shock	30.28	<5	63.11	6.25 (3.67–10.65)	<0.001
Disseminated Intravascular Coagulation and Shock	-	-	-	2.44 (1.67–3.56)	<0.001
ICU-level care	34.2	<5	70.88	2.00 (1.69–2.36)	<0.001

Legend: A dash (–) in the frequency columns indicates that the variable was constructed as an interaction term used exclusively for confounder adjustment in the mortality model and does not have a discrete prevalence estimate applicable to the descriptive cohort.

**Supplementary Table 3:** Factors associated with mortality in colon cancer

	**Sepsis % (n=107,230)**	**Non-Sepsis % (n=743,890)**	**Mortality % (n=18,990)**
Comorbidities			
Arrhythmia	12.13	8.36	15.17
Chronic Kidney Disease	13.89	9.1	16.09
Obesity	6.76	6.29	6.4
Palliative Care	22.55	8.41	51.45
Infections			
Gram-Negative Sepsis	6.12	-	6.85
E. coli	6.44	-	<5
Pseudomonas	<5	-	<5
Enterococcus	<5	-	<5
S. aureus	<5	-	<5

Legend: A dash (–) in the column indicates that the variable was not studied in the non-sepsis cohort.

Figures 6A–6B show the incremental charges and length of stay associated with sepsis in colon cancer.

**Figure 6A.**
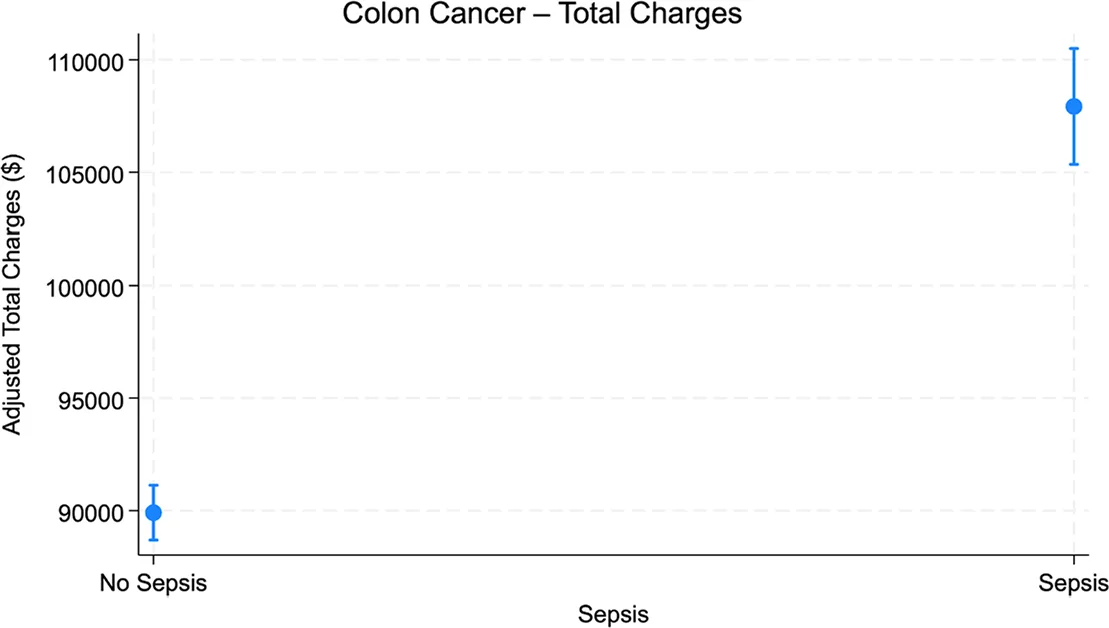
Total Hospitalization charges associated with sepsis in colon cancer Legend: Difference in total hospitalization charges among patients with colon cancer. Sepsis was associated with an increase in hospital charges of $18,017.33 (95% confidence interval $15,608.34–$20,426.32), estimated using propensity score–matched cohorts with multivariable adjustment.

**Figure 6B.**
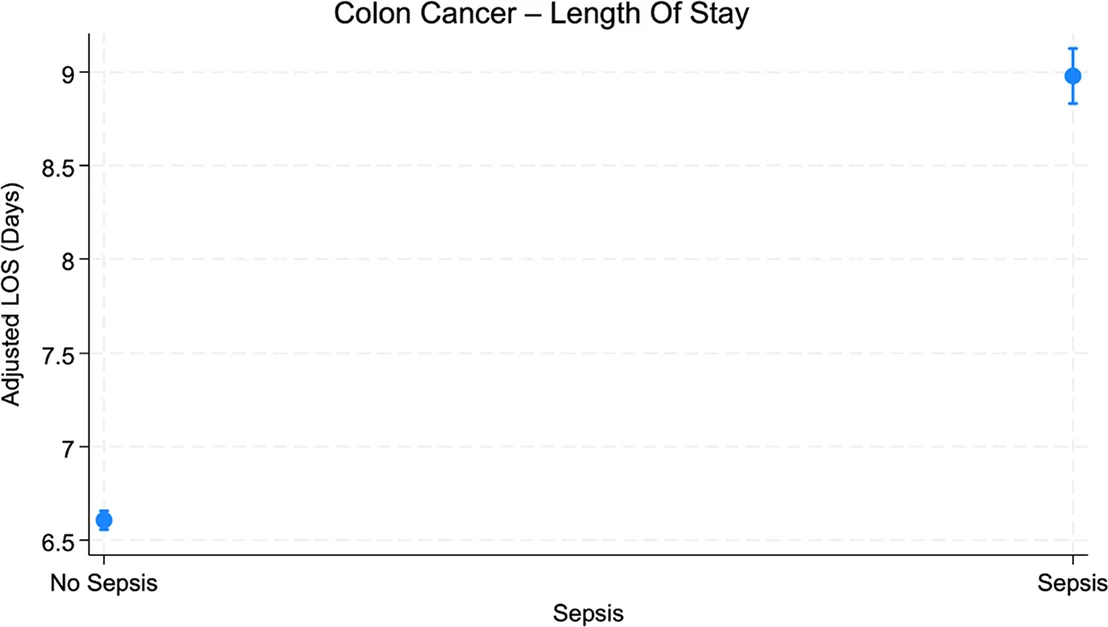
Length of stay associated with sepsis in colon cancer Legend: Difference in hospital length of stay among patients with colon cancer. Sepsis was associated with an increased length of stay of 2.37 days (95% confidence interval 2.22–2.52), based on propensity score–matched cohorts with multivariable adjustment.

### Impact of Sepsis in Hepatic Malignancy

Among 433,145 hospitalizations for hepatocellular cancer, 18.76% were admitted with sepsis. In-hospital mortality was markedly higher with sepsis than without sepsis (22.34% vs. 6.22%, p≤0.001). After propensity matching and adjustment, sepsis remained independently associated with mortality (aOR 1.60, p≤0.001), incremental charges of $15,725 (p≤0.001), and an additional length of stay of 1.93 days (p≤0.001).

Patients with sepsis were slightly older than those without sepsis (mean age 66.89 vs. 66.17 years). Females comprised a larger proportion of the sepsis cohort than the non-sepsis cohort. By race, both White and African American patients comprised slightly smaller proportions of the sepsis group than the non-sepsis group.

Among patients with hepatocellular carcinoma and sepsis, acute kidney injury (OR 2.16) and hepatic dysfunction (OR 2.08) were the leading organ-failure-related predictors of mortality, followed by metabolic encephalopathy (OR 1.44). Among complications, invasive ventilation (OR 7.66), acute respiratory failure (OR 3.43), shock (OR 2.67), ICU-level care (OR 1.86), and disseminated intravascular coagulation (OR 1.75) (all p≤0.05) were each independently associated with mortality. Table 4 and supplementary table 4 detail mortality predictors in hepatic malignancy.

**Table 4:** Factors associated with mortality in hepatic cancer

	**Sepsis % (n=81,245)**	**Non-Sepsis % (n=351,900)**	**Mortality % (n=18,145)**	**Adjusted OR (95% CI)**	**p-value**
Sociodemographics					
Age Over 60 years	75.08	73.24	74.51	1.01 (0.93–1.09)	0.888
Gender					
Male	64.98	67.21	68.26	Reference	Reference
Female	35.02	32.79	31.74	0.91 (0.84–0.97)	0.008
Race					
White	58.47	60.31	55.44	Reference	Reference
Black	12.57	13.81	14.95	1.18 (1.07–1.31)	0.001
Hispanic	16.4	15.58	16.08	0.93 (0.84–1.03)	0.144
Asian or Pacific Islander	7.96	5.94	7.62	1.20 (1.04–1.38)	0.01
Native American	<5	<5	<5	1.22 (0.85–1.75)	0.277
Other	<5	<5	<5	1.27 (1.05–1.52)	0.012
Hospital size according to the number of beds					
Small	15.71	16.26	14	1.16 (1.05–1.29)	0.005
Medium	26.94	24.97	26.76	1.12 (1.03–1.22)	0.012
Large	57.35	58.77	59.24	Reference	Reference
Comorbidites					
Diabetes	35.38	37.64	31.3	0.78 (0.72–0.84)	<0.001
Anemia	45.15	36.84	38.66	0.73 (0.68–0.78)	<0.001
Acute Organ Dysfunction					
Acute Kidney Injury	49.66	27.49	75.92	2.16 (2.01–2.32)	<0.001
Infective Endocarditis	<5	<5	<5	0.65 (0.49–0.85)	0.001
Metabolic Encephalopathy	15.26	<5	25.41	1.44 (1.30–1.60)	<0.001
Hepatic Dysfunction	24.03	20.69	43.37	2.08 (1.93–2.25)	<0.001
Myocardial Infarction	20.4	19.4	21.3	0.90 (0.83–0.99)	0.024
Complications					
Acute Respiratory Failure # Invasive					
Ventilation					
Acute Respiratory Failure	25.71	7.83	55.69	3.43 (3.14–3.76)	<0.001
Invasive Ventilation	12.78	<5	38.77	7.66 (6.22–9.44)	<0.001
Acute Respiratory Failure and Invasive	-	-	-	6.01 (5.24–6.90)	<0.001
Ventilation					
Disseminated Intravascular Coagulation # Shock					
Disseminated Intravascular Coagulation	<5	<5	7.55	1.75 (1.50–2.05)	<0.001
Shock	31.07	<5	64.56	2.67 (1.77–4.04)	<0.001
Disseminated Intravascular Coagulation and Shock	-	-	-	4.27 (3.03–6.02)	<0.001
ICU-level care	35.21	<5	72.97	1.86 (1.58–2.18)	<0.001

Legend: A dash (–) in the frequency columns indicates that the variable was constructed as an interaction term used exclusively for confounder adjustment in the mortality model and does not have a discrete prevalence estimate applicable to the descriptive cohort.

**Supplementary Table 4:** Factors associated with mortality in hepatic cancer

	**Sepsis % (n=81,245)**	**Non-Sepsis % (n=351,900)**	**Mortality % (n=18,145)**
Comorbidities			
Chronic Kidney Disease	14.83	13.28	18.32
Obesity	5.48	<5	6.39
Arrhythmia	9.3	7.02	11.02
Palliative Care	28.12	17.04	56.1
Infections			
Gram-Negative Sepsis	9.53	-	10.31
E. coli	8.03	-	5.04
Pseudomonas	1.46	-	<5
Enterococcus	5.82	-	<5
S. aureus	5.21	-	5.13

Legend: A dash (–) in the column indicates that the variable was not studied in the non-sepsis cohort.

Figures 7A–7B show the incremental charges and length of stay associated with sepsis in hepatocellular cancer.

**Figure 7A.**
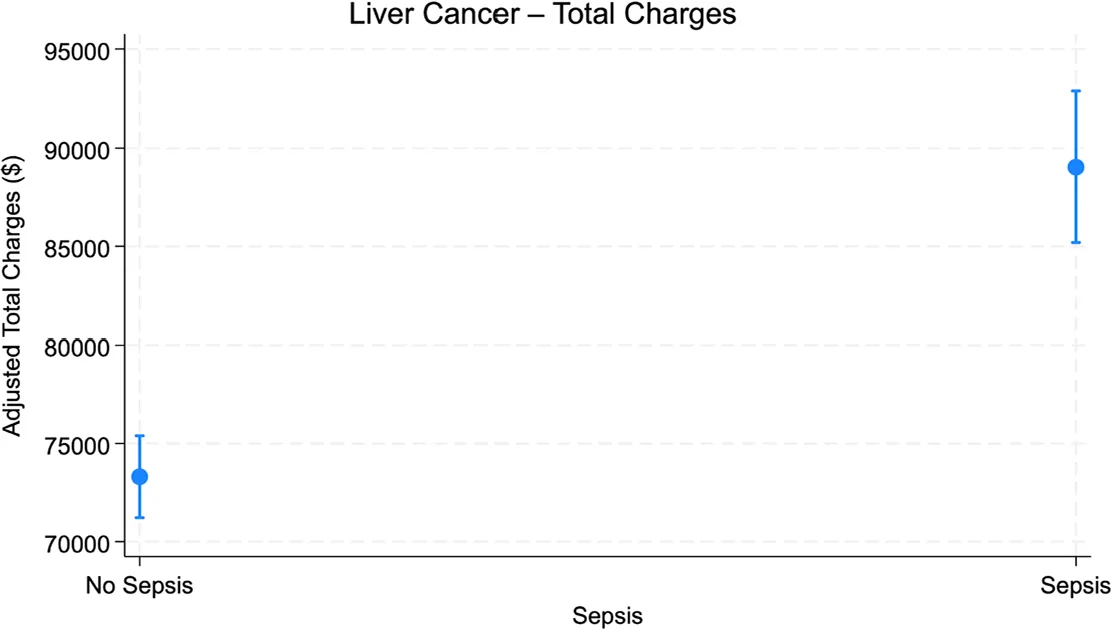
Total hospitalization charges associated with sepsis in hepatocellular cancer Legend: Difference in total hospitalization charges among patients with hepatocellular cancer. Sepsis was associated with increased hospital charges of $15,725.13 (95% confidence interval $12,379.23–$19,071.03), estimated using propensity score–matched cohorts with multivariable adjustment.

**Figure 7B.**
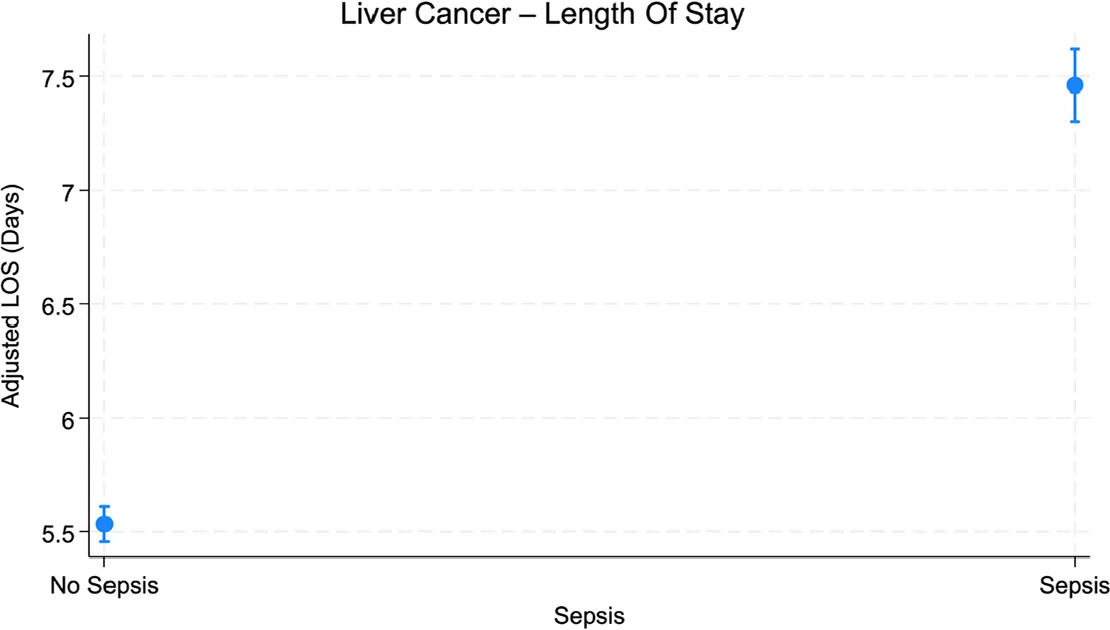
Length of stay associated with sepsis in hepatocellular cancer Legend: Difference in hospital length of stay among patients with hepatocellular cancer. Sepsis was associated with an increased length of stay of 1.93 days (95% confidence interval 1.76–2.09), based on propensity score–matched cohorts with multivariable adjustment.

### Impact of Sepsis in Pancreatic Malignancy

Among 557,204 hospitalizations for pancreatic cancer, 19.22% were admitted with sepsis. In-hospital mortality was markedly higher with sepsis than without sepsis (17.26% vs. 5.11%, p≤0.001). After propensity matching and adjustment, sepsis remained independently associated with mortality (aOR 1.43, p≤0.001), incremental charges of $9,351 (p≤0.001), and an additional length of stay of 1.23 days (p≤0.001).

Patients with sepsis were marginally older than those without sepsis (mean age 68.49 vs. 68.38 years). Females comprised a smaller proportion of the sepsis cohort than the non-sepsis cohort. By race, White patients comprised a smaller proportion of the sepsis group than the non-sepsis group, while African American patients comprised a slightly larger proportion.

Among patients with pancreatic cancer and sepsis, hepatic dysfunction conferred the highest organ-failure-related mortality risk (OR 2.88), followed by acute kidney injury (OR 2.21) and metabolic encephalopathy (OR 1.55). Among complications, invasive ventilation (OR 10.39), shock (OR 5.14), acute respiratory failure (OR 3.36), ICU-level care (OR 2.04), and disseminated intravascular coagulation (OR 1.57) (p≤0.05) were each independently associated with mortality. Table 5 and supplementary table 5 detail mortality predictors in pancreatic malignancy.

**Table 5:** Factors associated with mortality in pancreatic cancer

	**Sepsis % (n=107,130)**	**Non-Sepsis % (n=450,075)**	**Mortality % (n=18,485)**	**Adjusted OR (95% CI)**	**p-value**
Sociodemographics					
Age Over 60 years	79.22	77.21	79.77	1.121 (1.032–1.218)	<0.001
Gender					
Male	56.38	51.26	54.1	Reference	Reference
Female	43.62	48.74	45.9	1.113 (1.045–1.185)	0.001
Race					
White	68.43	71.39	62.67	Reference	Reference
Black	14.16	13.78	18.44	1.221 (1.113–1.339)	<0.001
Hispanic	9.38	8.3	9.47	1.067 (0.949–1.199)	0.278
Asian or Pacific Islander	<5	<5	<5	1.253 (1.073–1.462)	0.004
Native American	<5	<5	<5	1.704 (1.122–2.589)	0.012
Other	<5	<5	<5	1.381 (1.144–1.667)	0.001
Hospital size according to the number of beds					
Small	17	16.31	16.04	1.204 (1.084–1.337)	0.001
Medium	26.85	25.44	27.94	1.138 (1.053–1.231)	0.001
Large	56.15	58.25	56.02	Reference	Reference
Comorbidites					
Diabetes	42.84	40.88	37.92	0.74 (0.69–0.79)	<0.001
Anemia	48.91	34.57	41.71	0.72 (0.67–0.77)	<0.001
Acute Organ Dysfunction					
Acute Kidney Injury	37.93	17.22	66.38	2.21 (2.06–2.37)	<0.001
Infective Endocarditis	3.04	<5	<5	0.75 (0.58–0.97)	0.028
Metabolic Encephalopathy	11.48	<5	20.99	1.55 (1.39–1.73)	<0.001
Hepatic Dysfunction	7.15	<5	19.5	2.88 (2.56–3.24)	<0.001
Myocardial Infarction	21.14	19.13	24.45	1.04 (0.96–1.128)	0.334
Complications					
Acute Respiratory Failure # Invasive					
Ventilation					
Acute Respiratory Failure	20.64	5.99	49.34	3.36 (3.08–3.66)	<0.001
Invasive Ventilation	8.11	<5	30.35	10.39 (8.17–13.21)	<0.001
Acute Respiratory Failure and Invasive Ventilation	-	-	-	5.97 (5.19–6.88)	<0.001
Disseminated Intravascular Coagulation # Shock					
Disseminated Intravascular Coagulation	<5	<5	6.19	1.57 (1.33–1.85)	<0.001
Shock	26.68	<5	60.1	5.14 (3.75–7.05)	<0.001
Disseminated Intravascular Coagulation and Shock	-	-	-	3.54 (2.44–5.14)	<0.001
ICU-level care	29.54	<5	67	2.04 (1.72–2.43)	<0.001

Legend: A dash (–) in the frequency columns indicates that the variable was constructed as an interaction term used exclusively for confounder adjustment in the mortality model and does not have a discrete prevalence estimate applicable to the descriptive cohort.

**Supplementary Table 5:** Factors associated with mortality in pancreatic cancer

	**Sepsis % (n=107,130)**	**Non-Sepsis % (n=450,075)**	**Mortality % (n=18,485)**
Comorbidites			
Arrhythmia	10.13	7.4	11.71
Chronic Kidney Disease	9.97	8.16	13.47
Obesity	3.73	3.4	<5
Palliative Care	26.96	17.93	57.88
Infections			
Gram-Negative Sepsis	9.61	-	10.79
E. coli	9.09	-	6.09
Pseudomonas	1.26	-	<5
Enterococcus	5.41	-	<5
S. aureus	3.73	-	<5

Legend: A dash (–) in the column indicates that the variable was not studied in the non-sepsis cohort.

Figures 8A–8B show the incremental charges and length of stay associated with sepsis in pancreatic cancer.

**Figure 8A.**
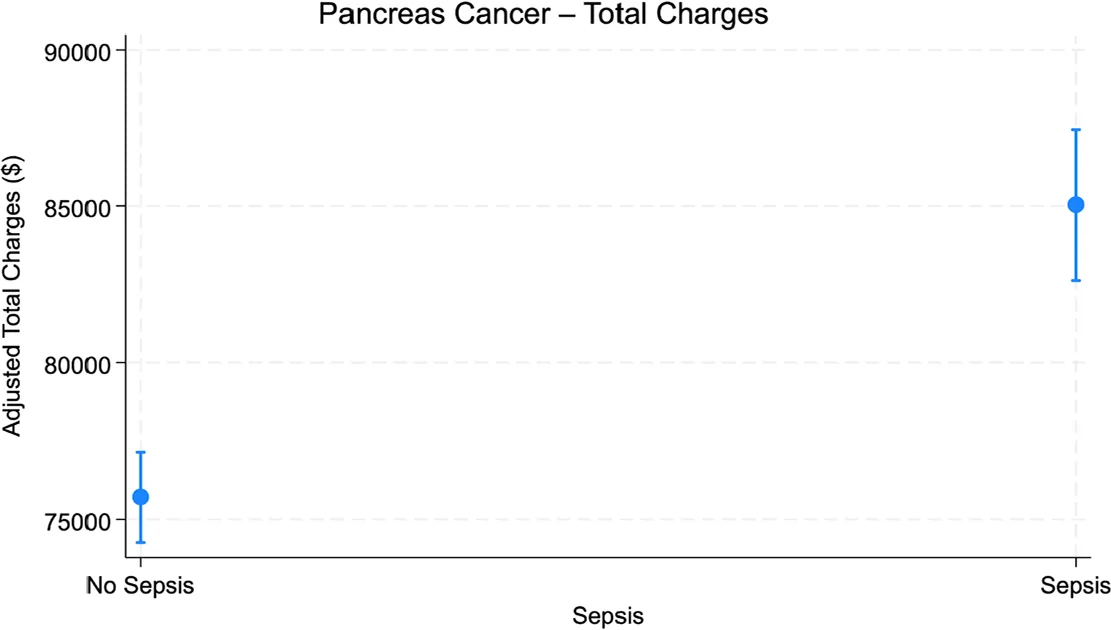
Total hospitalization charges associated with sepsis in pancreatic cancer Legend: Difference in total hospitalization charges among patients with pancreatic cancer. Sepsis was associated with increased charges of $9,351.10 (95% confidence interval $7,410.74–$11,291.46), estimated using propensity score–matched cohorts with multivariable adjustment.

**Figure 8B.**
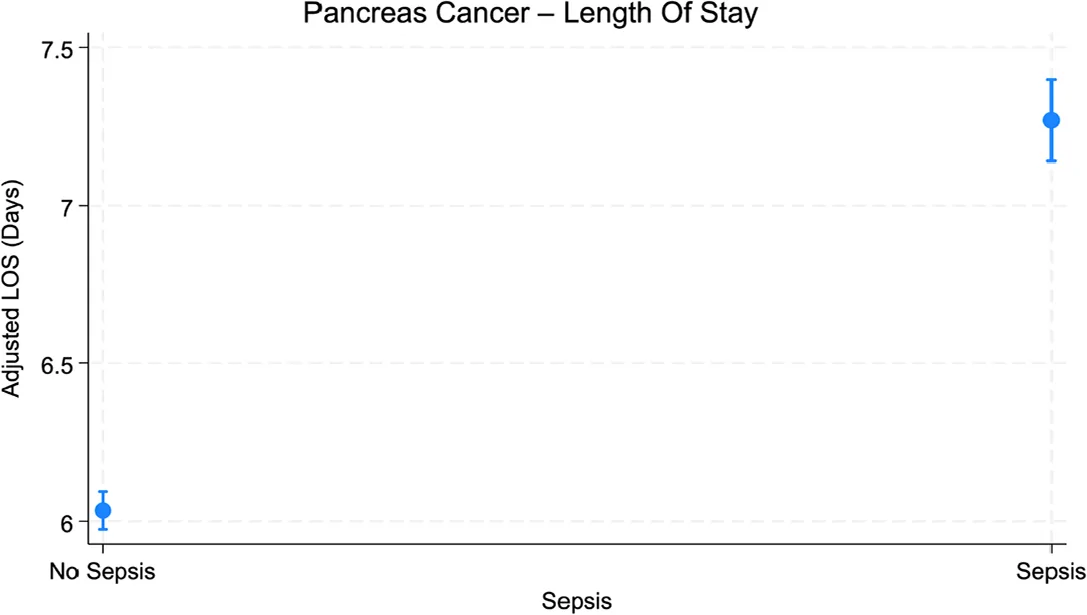
Length of stay associated with sepsis in pancreatic cancer Legend: Difference in hospital length of stay among patients with pancreatic cancer. Sepsis was associated with an increased length of stay of 1.23 days (95% confidence interval 1.11–1.36), based on propensity score–matched cohorts with multivariable adjustment.

## Discussion

To our knowledge, our study provides a comprehensive nationally representative analysis of sepsis outcomes across multiple gastrointestinal malignancy subtypes. We found notable variation in both sepsis prevalence and mortality across subtypes - hepatic and pancreatic cancers showed the highest sepsis prevalence (18.76% and 19.22%), while gastric and hepatic cancers carried the highest in-hospital mortality (23.57% and 22.34%). The mortality rate among patients requiring ICU-level care was highest in hepatic cancer (72.97%), followed by colonic (70.88%), gastric (70.28%), and pancreatic cancers (67%).

Patients with gastrointestinal malignancies are at increased risk of sepsis [17], especially after surgical procedures [18,19]. Several factors contribute to this, including immunosuppression from cancer and treatment with chemotherapy and immunotherapy, as well as invasive procedures such as central venous catheters, urinary catheters, ports, and drains [7,20]. Patient-specific factors, such as age, malnutrition, and overall health condition, play a role [21]. The study population was predominantly over the age of 60 years, and Liu et al. demonstrated that elderly patients have higher rates of sepsis among those with gastrointestinal malignancies [22]. The increased risk may be due to alterations in the host response, particularly in neutrophil chemotaxis, adhesion, and intracellular killing, as well as defects in humoral immunity, including both antibody responses and complement opsonization [23]. These patients also tend to have a higher burden of comorbidities. Our study found that diabetes mellitus was associated with significantly lower odds of mortality among patients with gastrointestinal malignancies. Similarly, a meta-analysis by Xiang et al. reported that diabetes mellitus was not significantly associated with increased mortality [24]. We believe that while diabetes may contribute to the occurrence of sepsis, it does not appear to play a major role in influencing mortality outcomes in this population.

Edwards et al. showed that in patients with cancer and sepsis, the overall mortality among patients with cancer and sepsis was 54%, with ICU-level mortality reported at 44%. Our findings among patients with gastrointestinal malignancies revealed a lower total mortality rate (17.26–23.57%), but a markedly higher ICU-related mortality (67–72.97%) [25]. These comparisons should be interpreted with caution, as differences in patient populations, including cancer stage, performance status, sepsis definitions and comorbidity burden may substantially influence reported mortality figures. A 2017 Korean population-based study by Kim et al. showed that mortality from septic shock in cancer patients decreased from 55.2% in 2009 to 47.8% in 2017 (p<0.001) [26], while our results indicate a higher mortality in patients with gastrointestinal malignancies who developed shock (60.1–64.56%), though differences in database methodology and population characteristics limit direct comparison. We believe that in gastrointestinal malignancies, once critical illness develops, outcomes may be disproportionately worse than those of other forms of cancer. Mirouse et al. described that malignancy-associated sepsis-like syndromes, such as disseminated intravascular coagulation (DIC), cytokine storm, acute respiratory distress syndrome, and tumor lysis syndrome, can occur, and they are common mimickers of sepsis [27]. Our results indicate that, the occurance of acute kidney injury, DIC, shock, or respiratory failure, a higher risk of mortality persists. Hence, these patients may benefit from specialized expertise and management protocols developed by intensivists, pulmonologists, nephrologists, oncologists, or hematologists for the management of cancer-related sepsis [28,29]. These patients may benefit from being treated in oncology-specific units or earlier admission to intensive care, given the higher level of care required [25,30,31,32,33]. Patients requiring 3 or more days in the ICU may benefit from a palliative care consult, but prospective studies are required to investigate this [34].

Limitations of our study include data primarily obtained from clinical registries using billing codes, discharge diagnosis codes, and other similar identifiers, which introduce the possibility of coding errors. We lack the laboratory values necessary for precisely diagnosing sepsis. Information on the patient’s chemotherapy regimen, overall staging, or histologic grading of tumors could not be identified. The study also lacks control over unidentified confounding variables. Evidence regarding the initiation of therapy and the persistence and gravity of complications is scarce. Since the data is derived from clinical registries, we were unable to identify the patient’s code status, goals of care, or palliative care intent at the time of admission. Future studies focusing on prophylactic measures and treatment perspectives on current guideline-directed therapies and the impact of early palliative care integration in this population are needed. Machine learning applications in clinical settings are yet to be explored [35,36].

## Conclusions

In our study, we investigated the outcomes of sepsis among adults in the United States with gastrointestinal malignancies, including gastric, pancreatic, hepatic, and colonic cancers, using data from the Healthcare Cost and Utilization Project. Our analysis revealed that patients with gastrointestinal malignancies experience high mortality rates when affected by sepsis, with acute organ dysfunction and the need for ICU-level care identified as key predictors of poor outcomes. Patients with sepsis had higher palliative care utilization, hospitalization costs, and a longer length of stay. These findings underscore the need for further prospective studies to evaluate optimal management strategies and the role of early palliative care integration in this high-risk population.
